# Exploring reasons for sick leave due to common mental disorders from the perspective of employees and managers – what has gender got to do with it?

**DOI:** 10.1080/17482631.2022.2054081

**Published:** 2022-03-27

**Authors:** Lisa Holmlund, Helena Tinnerholm Ljungberg, Ute Bültmann, Kristina Holmgren, Elisabeth Björk Brämberg

**Affiliations:** a Institute of Environmental Medicine, Unit of Intervention and Implementation Research for Worker Health, Karolinska Institutet, Stockholm, Sweden; b Department of Health Sciences, Community and Occupational Medicine, University of Groningen, University Medical Center Groningen, Groningen, The Netherlands; c Institute of Neuroscience and Physiology, Department of Health and Rehabilitation, Sahlgrenska Academy, University of Gothenburg, Gothenburg, Sweden

**Keywords:** Adjustment disorder, anxiety disorders, depression, occupational health, occupational science, sickness absence, social norms, gender roles, workplace, work-life balance

## Abstract

**Purpose:**

The purpose of this study was to explore the employee and the managerial experience of reasons for sick leave due to CMDs in relation to work and private life, through the lens of a transactional perspective of everyday life occupation and gender norms.

**Methods:**

Semi-structured interviews were conducted with 17 employees on sick leave due to CMDs and 11 managers. By using transactional and gender perspectives in a reflexive thematic analysis, themes were generated in a constant comparative process.

**Findings:**

Four themes were identified: a) struggling to keep up with work pressure and worker norms; b) struggling with insecurity in an unsupportive work environment; c) managing private responsibilities through flexible work schedules, and d) managing emotions alongside unfavourable working conditions.

**Conclusion:**

Sick leave due to CMDs was understood as related to experiences of accumulated events situated in different social, cultural, and societal contexts of everyday life. Practices and policies should encourage an open dialogue about work and private life and health between employees and managers. To build healthy and sustainable work environments practices should also aim for increased awareness of social norms. A better understanding may facilitate the identification of situations in work and private life that are problematic for the employee.

## Introduction

Understanding the reasons for sick leave due to common mental disorders (CMDs, i.e., mild to moderate depression, anxiety, adjustment, and stress-related disorder) is important because of the great burden posed by sick leave (Organisation for Economic Co-operation and Development (OECD), 2015). It is also important in terms of equality because of the gender gap, with more women than men reporting sick (Mastekaasa & Melsom, 2014). In 2019, mental disorders accounted for 53% of all sick leave cases for women in Sweden, and 42% of all sick leave cases for men (The Swedish Social Insurance Agency (SSIA), 2020).

Factors related to the individual and the workplace are the more commonly described reasons for sick leave. Individual-level reasons, refer to factors such as symptom severity and low educational level, and work-related reasons to high job demand in combination with low control and low support (Holmgren & Dahlin Ivanoff, 2004; Verdonk et al., 2008; De Vries et al., 2018). Moreover, there is an increasing interest in the negative impact of work-home interference on mental health and well-being (Blom et al., 2014; Neto et al., 2018) and on sick leave due to CMDs (Svedberg et al., 2018). However, the integrated effect of work and private life for sick leave due to CMDs is less described. Using a gender perspective in the exploration of this type of sick leave can generate knowledge of the complexities of everyday life and health (Connell, 2012; Heise et al., 2019; Wilcock & Hocking, 2015). In this endeavour, exploring the combined perspectives of employees and managers is relevant because of the impact of workplace and leadership support for worker health and well-being (M. B. Nielsen et al., 2013; Holmgren & Dahlin Ivanoff, 2007; K. Nielsen et al., 2018).

Gender segregation and the double burden hypothesis have been in focus in research addressing the gender gap in sick leave. Gender segregation refers to the gendered division of labour (horizontal gender segregation) and the gendered opportunities for career progression (vertical gender segregation) (Charles, 2003). The double burden hypothesis states that the combination of burdens at work and in private life can increase the risk of sick leave (Bratberg et al., 2002; Nilsen et al., 2017). Overall, the larger share of domestic work carried out by women (Cunha et al., 2016) is one explanation for the gender differences in sick leave. Associations between the double burden and sick leave are described for both genders, including those on sick leave due to CMDs. However, to date, there is insufficient evidence to support the contention that the double burden hypothesis explains the gender gap in sick leave. The lack of evidence might reflect the complexity of measuring domestic work, for example, the distress arising from perceived injustice or dissatisfaction with the division of work (Nilsen et al., 2017; Staland-Nyman et al., 2021; Svedberg et al., 2018).

Using a gender perspective in exploring reasons for sick leave in relation to work and private life in a Swedish setting is relevant for several reasons. Swedish research shows a long-term trend towards high strain jobs, especially among care workers (Aronsson et al., 2021), and an increased risk of sick leave due to mental disorders in female-dominated sectors, such as healthcare, education, and social services (Lidwall et al., 2018). Sick leave patterns due to mental disorders show an increased risk for women, persons aged 30–39, and parents with underaged children (Lidwall et al., 2018). Moreover, Sweden is consistently ranked high in gender equality assessments, and labour market participation is nearly equal among men and women (European Institute for Gender Equality (EIGE), 2020). However, horizontal gender segregation and a pay gap remain (Hustad et al., 2020; Swedish National Mediation Office, 2021). In addition, Swedish women report a lower level of gender equality than men (Staland-Nyman et al., 2021), and inequality in domestic work persists despite policy measures, such as parental leave (Eydal et al., 2015).

Qualitative research into gendered experiences of sick leave due to CMDs indicates that it is a complex, downward process for women. Verdonk et al. (2008) describe an interactive process between women’s needs and worker norms in which emerging symptoms of CMDs develop and reporting sick is a “last resort”. Holmgren and Dahlin Ivanoff found that sick leave resulted from a “process of losing control” in everyday life (2004, p. 216). This process involved an interplay between individual attributes (e.g., high sense of responsibility and/or difficulties in setting limits) and workplace-related factors (e.g., indistinct organization and/or conflicts). To our knowledge, less qualitative research has explored men’s subjective experience of reasons for sick leave and reasons related to private life. In a study of return-to-work (RTW), women experienced having extensive practical responsibilities and providing emotional support as barriers to RTW after sick leave due to CMDs. For men, private life was often perceived as an arena for emotional support facilitating RTW, while the struggle to achieve and uphold social norms of masculinity was described as a barrier (Nybergh et al., 2020). Struggling to uphold social norms of masculinity is also described in relation to men’s experience of CMDs (Drioli-Phillips et al., 2020; Scholz et al., 2017).

To understand the richness and complexities of everyday life and how this relates to worker health and wellbeing, we argue that it is important to use a qualitative approach and explore what people do and experience in their everyday lives and how this is situated, i.e., shaped, embedded, and negotiated in relation to social, cultural, and societal contexts (Rudman, 2010; Wilcock & Hocking, 2015). In addition to previous studies, it is important to include the experiences of male employees and the manager’s perspective (cf., M. B. Nielsen et al., 2013; Drioli-Phillips et al., 2020; Holmgren & Dahlin Ivanoff, 2007; K. Nielsen et al., 2018; Nybergh et al., 2020; Scholz et al., 2017; De Vries et al., 2018). Therefore, the purpose of this study was to explore the employee and the managerial experience of reasons for sick leave due to CMDs in relation to work and private life. This will be explored through the lens of a transactional perspective of everyday life occupation and gender norms.

### Theoretical perspectives

In this study, we regard everyday occupation and health from a transactional and a gender perspective (cf., Connell, 2012; Dickie et al., 2006; Fritz & Cutchin, 2017; Heise et al., 2019; Wilcock & Hocking, 2015). The transactional perspective implies that what people do and experience in everyday life is embedded in and shaped by social, cultural, and societal contexts, and contributes to their health and well-being. From a transactional perspective on occupation, everyday life is viewed as loaded with possibilities or problematic situations that require attention, contemplation, and action. As such, a person’s responses to the problematic dimensions of everyday life are viewed as integral to the situation that generates them. This perspective gives an ongoing and dynamic understanding of everyday occupations (Fritz & Cutchin, 2017). Situations can be understood as “where and when the transactions happen” (Rosenberg & Johansson, 2013, p. 147) and it should be recognized that multiple transactions (person-situation relations) can be present in one situation (Dickie et al., 2006).

According to the gender theoretical perspective of the study, men and women are positioned in a hierarchical (but changeable) structure (Connell, 2012; Landstedt et al., 2009). In this structure, the burden of gender inequality first and foremost affects women’s health, while restrictive gender norms might affect the health of men, women, and gender minorities negatively (Heise et al., 2019). From a gender perspective, the ongoing person-situation relation pertains to the notion of the (re)creation of what it means to be a man or a woman as a process of “doing gender”, in which individuals either conform to gender norms or deviate from them in everyday practices (West & Zimmerman, 1987). Thus, people “do gender” in specific social and cultural contexts that have different demands and expectations of men and women respectively (Magnusson & Marecek, 2012, p. 34). The inability to fulfil these perceived and at times competing gendered expectations in work and private life may give rise to feelings of insufficiency and ill-health (Vidman, 2007). By focusing our study on the experience of sick leave due to CMDs, we highlight the intersection between work, private life, and health.

## Methods

This study used individual semi-structured interviews that were analysed using a reflexive thematic approach (Braun & Clarke, 2006, 2014, 2019). Ethical approval was obtained from the Swedish Ethical Review Authority (reference numbers 496–17; 2020–02462). The reporting follows the consolidated criteria for reporting qualitative research by Tong et al. (2007). The authors represent a multidisciplinary group with experience in research related to sick leave due to CMDs, everyday life occupations, and gender perspectives.

### Participants and procedure

The employees in the present study were all included in a randomized controlled trial (RCT) evaluating the effectiveness of a problem-solving intervention among employees on sick leave due to CMDs (PROSA; Björk Brämberg et al., 2018). Eligibility criteria for the RCT were as follows: age 18–59; sick leave of 2‒12 weeks due to mild to moderate depression, anxiety, or adjustment disorder; accepting the involvement of their manager. Exclusion criteria were: severe depression; other severe mental disorders (e.g., psychotic or bipolar disorders, or referral to a psychiatrist); pregnancy; somatic complaints or disorders that affect workability; inability to read, write and understand Swedish. The eligibility criterion for managers was that they should be responsible for the rehabilitation of a participant included in PROSA.

From the original sample (Björk Brämberg et al., 2018), a purposive sampling relating to the employees’ gender, age, educational level, and work sector was used to generate a broad range of experiences. Twenty-eight participants were recruited (17 employees and 11 managers), of whom 18 were included in employee-manager pairs. Inclusion was initiated by a first contact by telephone between employees and the first author, in which 20 employees were informed about the study purpose and procedure. For those 18 who expressed interest (two declined participation in the interview) further information about the study and procedure was shared and they had a possibility of posing questions to the researcher. Thereafter written information was sent upon consent, and the employees were asked to agree to the researcher contacting their manager. One employee cancelled the interview after giving consent. Eight employees did not want their manager to be contacted, either for privacy reasons or because the employee or the manager had changed jobs. Accordingly, nine managers were contacted after the employee interviews. Information and consent followed the same procedure as for the employees. All nine managers agreed to participate. Another round of three managers was contacted with the consent of their employees; two of them agreed to participate. Seven managers were first-line managers, one was a chief executive officer, and one was a school principal. All had rehabilitation responsibilities for an employee included in PROSA. Demographic characteristics are presented in Table I.

**Table I.** Participants’ demographic characteristics

			Employees		Managers	
		Men	Women	Men		Women
Number		8	9	4		7
Age, *years, mean (range)*		46 (24–55)	44 (34–54)	49 (36–63)		44 (32–54)
Level of education^1^						
Primary/ secondary education		5	4	1		0
Higher education/ university		3	5	2		6
Work sector						
Private sector		7	4	4		3
Municipality or regional sector		1	5	0		4
Work experience						
< 2		4	4	1		0
3–10		2	2	2		5
>10		2	3	1		2
Living with a partner, *yes*		7	7	NA		NA
Children living at home, *yes*		4	7	NA		NA
Occupational status, *permanent employment*		6	8	NA		NA
Current sick leave, *yes*		2	0	NA		NA
Diagnoses for sick leave, stress/ depression/ anxiety & depression^2^				NA		NA
Adjustment and stress-related disorders		4	7			
Depressive disorder		3	2			
Anxiety disorders		1	0			
Changes at work after RTW				NA		NA
Part-time work		0	3			
Change of occupation (employment or education)		1	1			

NA = Not applicable

^1^Missing data for two managers

^2^Main diagnosis as the reason for sick leave, collected from The Swedish Social Insurance Agency’s register Micro Data for the Analysis of Social Insurance register (MiDAS).

### Data generation

Semi-structured interviews were held between October 2020 and January 2021. Due to the Covid-19 pandemic, interviews were carried out by telephone. All were conducted by the first author (PhD) with previous experience in qualitative interviewing. The interviews were conducted at a time convenient for the participant and lasted 40‒100 minutes. All participants were encouraged to find a space where they felt they could speak freely and if possible be undisturbed. Two interview guides were developed in the research group based on the purpose of the study and research literature (Nybergh et al., 2020). One was for employees, the other for managers. The interview guides had three conceptual areas: 1) the employees’ everyday life before sick leave/ the managers’ perception of the workplace and workplace culture; 2) perceived reasons for sick leave related to work life and private life; 3) the influence of social, cultural, and societal norms on sick leave. Examples of questions to the employee were: “Can you tell me about your everyday life today, how is it different from before your sick leave?”; “What are your thoughts about why you had to go on sick leave?”; “Are there expectations in your work or private life that impact your work?” Examples of questions for the managers were: “Can you tell me about the workplace where you are the manager?”; “What are your thoughts about why employees at your workplace go on sick leave due to CMDs?”; “Based on what we have discussed, are there differences between employees at your workplace?” Follow-up questions and prompts were used to obtain rich descriptions, for example, by asking about specific situations or cases. All interviews were digitally recorded, transcribed verbatim, and checked for accuracy. Field notes were written after each interview.

### Data analysis

The interview transcripts were analysed following reflexive thematic analysis. This method highlights the centrality of researcher reflexivity and offers flexibility regarding the theoretical approach used (Braun & Clarke, 2006, 2019). The transactional and gender perspectives used in the analysis are outlined in the introduction. The theoretical perspectives helped search for relations between the person and social, cultural, and societal contexts in work and private life. Analysis was initiated by careful reading, coding, and discussions between the first and the second author. Coding focused on semantic and latent meanings. Examples of semantic coding were the employees’ and managers’ reasoning about workload and double burden preceding the sick leave, while latent meanings were coded when situations were framed in relation to social norms of work and gender. The fourth and the fifth author read several of the interview transcripts and contributed to these discussions. The analysis was constantly moved forward by asking questions about the data and looking for similarities and differences across codes to generate themes. The theoretical perspectives were helpful in this process and examples of questions are: how do employees and managers experience and respond to the problematic situations they experience and how do their stories relate to gendered norms? Themes were thus generated by the employee and managerial experience of certain situations relevant for the employees’ sick leave, such as ‘keeping up with work pressure and worker norms. All authors helped to review initial themes—i.e., scrutinizing if themes were coherent and represented an accurate description of the data—as well as defining and naming themes. As such, all steps involved a constant movement between the data, codes, and theme development and collaboration between co-authors (Braun & Clarke, 2006).

## Findings

**Figure 1.**
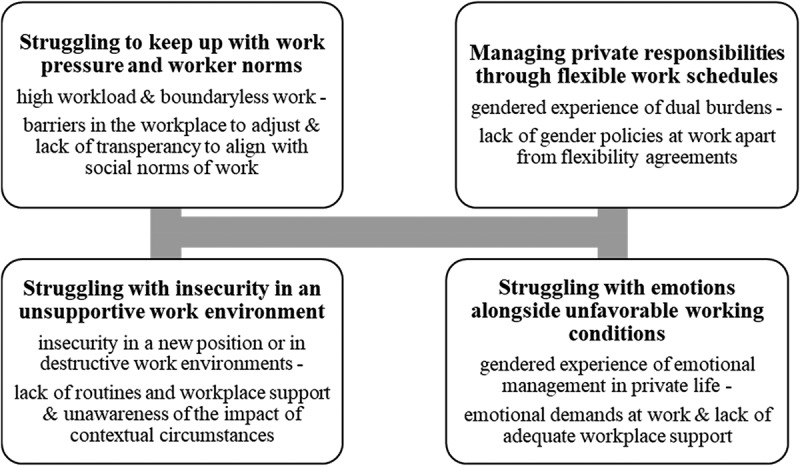
Four themes illustrate the experience of events in work and private life in relation to contextual circumstances and social norms of work and gender. Sick leave due to CMDs was related to the experience of accumulated events situated in different social, cultural, and societal contexts of everyday life.

Four themes were generated by our analysis: a) struggling to keep up with work pressure and worker norms, b) struggling with insecurity in an unsupportive environment, c) managing private responsibilities through flexible work schedules, and d) managing emotions alongside unfavourable working conditions (Figure 1). The themes establish events in work and private life contexts which are characterized by high workload and boundaryless work; insecurity at work; double burdens; and emotion management. The analytic storyline relates to employee and managerial experience of these events, and the positioning of their respective experiences to contextual circumstances, e.g., workplace organization, culture, and social norms of work and gender—and how this contributed to sick leave.

### Struggling to keep up with work pressure and worker norms

The first theme reflects employee perceptions of high workload and boundaryless work as reasons for their sick leave. Framing employees’ work experiences through context reveals a longstanding struggle where the needs of the employee are not sufficiently met or hidden to align with social norms of work.

Both men and women described situations in which high workload and boundaryless work were coupled with struggles of “not being able to finish” and keeping up with work tasks. High workload and boundaryless work were defined by unclear and overlapping work tasks, multiple contacts, and uncontrolled workflow (exemplified by a large number of emails, requests from colleagues, and unexpected changes in job assignments). The fact that workload could increase with little or no notice resulted in interruptions in the workflow. The struggle to keep up with work was felt, by both male and female employees to cause symptoms such as weariness, dizziness, sleeping problems, anxiety, and/or panic attacks, and was often perceived as the main reason for sick leave, illustrated by this employee:

I’m a union representative also, and safety representative, and I was responsible for the work schedule. So, it was difficult to do my caring duties and at the same time run around between a lot of meetings. I thought it was fun, but eventually, it came to a stop. Then I just had to sleep. Work, sleep, work, sleep, that’s all I did the last few weeks. [Female, 40 years, stress]

The heavier workload meant that the employee needed to work at maximum capacity during working hours with little or no time for a break. Their time off work was used for recovery. One employee said: “I always have that feeling that no matter how much I work, I will never finish, or do it well enough”. [Male, 49 years, depression] The employees’ stories show that tasks that initially felt fun, engaging, and rewarding, came to be imbued with a loss of control. Inability to make sense of work, because a perceived gap between the work performed and the work they would ideally like to perform, was a further source of emotional distress.

The downward process preceding sick leave also needs to be understood through organizational and cultural contexts. Firstly, managers’ inability to “turn off the tap” on their employees’ workload and inertness in organizations was seen as hindering the opportunities to adjust for a high workload and perpetuated a situation of intense work and little recovery. The sense of being stuck in a situation with no prospect of their work circumstances improving increased employees’ distress and could lead to disengagement from work. Secondly, ideals of being a good worker affected opportunities for improving the situation for employees. Some managers defined an ideal worker as someone who could keep up a high momentum at work, produce high-quality work, make sound priorities, stay healthy, and contribute to effectivisation. As one manager said:

We have a culture that breathes performance and high tempo. In a way, we are stretching the limits when it comes to pressure, stress, delivery, and so on. I have a lot of good people here that can manage large amounts of that, but in combination with having a difficult time at home … You can see when that’s happening. [Male, 36 years, private sector]

The ideal worker was in this theme understood in line with social norms of a masculine worker ideal—as someone not burdened by their private life (Acker, 1990). For both male and female employees, the worker ideal could mean that talking about their situation or going on sick leave posed the potential risk of moving from an appreciated position to being seen as “weak”. Inability on the part of the employee to fulfil the ideal worker norm contributed to the downward process leading to sick leave. Simultaneously, not communicating their difficulties seemed to result in worse and lengthier symptoms.

### Struggling with insecurity in an unsupportive work environment

In the second theme, feelings of insecurity in a new job position and in unsupportive, or even destructive, work environments were featured in both male and female employees’ stories about why they had to go on sick leave. Adequate support was constrained by power relations, the work culture, and social norms—and by the managers’ unawareness of these factors.

The insecurity caused by not feeling able to manage work tasks in a new job and experiencing unsupportive or destructive work environments gave rise to a feeling of personal inadequacy and/or of not being appreciated. For the employees, these experiences caused distress and gave rise to symptoms such as weariness, cognitive symptoms, anxiety, and rumination. In a downward process, the symptoms could contribute to further distress and sick leave. One employee exemplified this downward process when he engaged in a new job and his symptoms due to CMDs emerged:

I wanted to appear a bit competent, and when you don’t feel so competent, which was the case for me, this is what becomes difficult. That you want to show you can perform, but actually, you don’t have the energy. [Male, 45 years, depression]

The employee exemplifies how his distress progressed when experiencing that his work performance was impaired by weariness and cognitive symptoms. This downward process was also noted for those employees who felt unappreciated or caught up in workplace conflicts. One employee described her insecurity over the risk of conflicts with the operative manager: “You don’t know if you’ve done the right thing or not, and it’s almost like you don’t dare to press any button. If I press the wrong button, what happens next? Yes, then they gets angry” [Female, 36 years, depression]. Interviewees saw such situations as partly related to individual processes. Employees and managers described how employees got stuck in trying to find solutions, or that they could not shake off negative comments.

In addition, reasons were sought in organizational and leadership support. Lack of adequate routines and support when starting a new job meant “flying blind” and led to a certain sense of powerlessness which was commonly described by men. Organizational reasons were to some extent acknowledged among managers and all expressed an explicit desire to be supportive. However, they called for transparency from the employee to be able to provide the right support to stop the downward process or ease the conflict:

The employee also needs to be communicative with the company … if you feel ‘no, this isn’t working’ or ‘this is too much’ or suchlike, it needs to reach the right person, the line manager, if I’m to be able to make any changes. And then we do as much as we can. [Female, 36 years, private sector]

This transparency could be difficult for the employee. Because of power relations, employees juxtaposed disclosing their insecurity with possible (further) negative consequences for their position at work, and signals from the employee could therefore be subtle or non-existent. This theme entailed an interesting gender difference illustrated when managers were inattentive to early signs of employee stress and depression. There was a risk that efforts to communicate would shut down—something mainly expressed by the female employees. Among men, on the other hand, working in destructive work environments with a hard jargon, a more common response was to act out and go along with this jargon. The distress and rumination that often followed for the employee could fuel symptoms of CMDs and conflicts.

### Managing private responsibilities through flexible work schedules

The third theme reflects practical and mental domestic management and responsibilities which in “an unfortunate interplay” with work were described as reasons for sick leave. Flexible work schedules were an appreciated solution to juggling dual burdens but could also perpetuate social norms and lead to shift working and lack of recovery, especially for women.

While the dual practical responsibilities of work and private life were part of everyday for most employees, what was noticeable was how mental labour—“thinking performed for the sake of accomplishing family goals” (Robertson et al., 2019, p. 196)—was more common among female employees. For them, this mental labour was particularly difficult to “check out” from, by asking for or receiving help, when stress-related symptoms arose. One employee said: “You have that role of project leader at home, that I think many women have, that also affects you cognitively—and when your cognition is affected it’s kind of not very easy”. [Female, 47 years, stress] The cognitive strain in private life added to the stress she already felt in her work, and lacking possibilities to check out meant that there was limited time for active and passive recovery. Instead, women felt caught in a paradox of increasing weariness and impaired sleep—and feelings of personal inadequacy if they were unsuccessful in fulfiling social and work norms. Male employees who were experiencing emerging symptoms of CMDs spoke of more instant relief and being less burdened by expectations on themselves or from others in their private life compared to female employees.

The managers acknowledged burdens and/or gender inequalities in private life as a reason for sick leave. Some perceived the challenges chiefly in terms of ideals about what could be achieved after working hours, and/or commitments in private life that spilled over into work and caused distress. As such, gender norms were more apparent in managers’ accounts of their employee’s private life (particularly family life) than in relation to their workplaces and organization. In work life, managers pointed to the importance and benefits of gender balance in terms of having both female and male employees in their teams, otherwise, gender policies at work were notable for their absence (or were not actively used). Measures to enable a work-life balance were sometimes exemplified by individual arrangements for part-time work or job assignments, but a flexible work schedule was considered primarily to be an equality measure. One male manager spoke about such measures:

We have a majority of people with 10–15 years of experience, and then you’re in the category of having small children. Then a lot of it’s about adjusting to private life. We’ve had to take this into account when building up our business … / … lots of people working 75-80% [of full-time]. And yes, lots of flexibility about picking children up from nursery and school, taking them to school, that sort of thing. [Male, 48 years, private sector]

Implicit in a flexibility agreement was “as long as the work was done on time”, echoing an idea that flexible working times would facilitate a better balance between work and family. While flexible schedules were seen as helping people to juggle the responsibilities of work and private life, boundaryless work also meant that ideal worker and parent norms could prevail at the cost of recovery. After doing the “second shift” at home (Hochschild & Machung, [1989]2003), parents were inclined to resume work after putting the children to bed. Perpetuation of dual responsibilities impeded recovery and, with hindsight, was seen as one reason for sick leave, and was especially seen among women.

### Managing emotions alongside unfavourable working conditions

The fourth theme reflects how challenging life events in private life meant that the everyday was imbued with emotional distress and the need to provide emotional support for others, i.e., emotion work (Hochschild, [1983]2012). Emotional management in private life was more pronounced among women and was a reason for sick leave, coupled with unfavourable working conditions.

In one way, the life events illustrated by the employees were out of the ordinary, yet they were also events that anyone might have to face during their working life. These include being the parent of a child with special needs; grieving over the loss of a relative; having marital problems or poor family relations; or living with a spouse diagnosed with a CMD. One male employee described a long-term emotional struggle caused by the fact that his child had suffered from a severe illness. He talked about his everyday situation as a “ticking time bomb” due to fear of relapse for his child. A female employee, who was a single parent described a typical day before sick leave as follows:

Maybe I started early and had to begin the day with a fight to get my son up. And then, your body gets very anxious and stressed. And you get to work and are supposed to deal with a child that might kick and fight and call you all sorts of inappropriate things. And you have colleagues who close their eyes to it so as not to see when you need support. [Female, 40 years, burnout]

Because of their private life, the working day was full of insecurity, partly because of delays or interruptions, but mainly due to emotion work and emotional distress. Some participants perceived such events as the main reason behind their increased symptoms of CMDs and sick leave. However, many employees spoke of emotional distress and/or emotion work as part of the background to why they ended up on sick leave: they linked their private circumstances and their situation at work. Ongoing emotion management was felt to reduce employees’ resilience to unfavourable working conditions. As exemplified in the citation, feelings of personal inadequacy in multiple roles and lack of recovery during the day were pronounced when emotion work was intertwined with providing support for others in their profession, i.e., emotional labour (Hochschild, [1983]2012). Employees described how ongoing emotion management in combination with a lack of collegial and/or leadership support at work increased their distress.

Managers often said that they were aware of an employee’s circumstances and understood how this affected their performance, mood, or resilience to stress. While emotional management was seen as something human, the managers also spoke of how it could negatively affect work performance and the atmosphere at work. Just as with work demands, private matters affecting work needed to be communicated by the employee for the manager to be able to adjust for them. For the manager, the communication with the employee entailed a delicate balance in terms of privacy. One manager reasoned:

… you ask sort of ’how are things at home these days, do you feel it’s a lot’, or ‘is there anything you feel is overwhelming?’, ‘is there anything we can do?’ … / … But if the person doesn’t open up there and then, I feel that I don’t really have the right to be there. [Female, 48, private sector]

The stories show that while it was taken for granted that emotional and practical support should be available for an employee in situations of acute emotional distress (such as the death of a relative), adjusting for ongoing emotion work seemed more challenging. Additionally, due to the sensitivity of and stigma associated with some life-events, such as abuse or poor family relations, some life-events were not shared in a work setting. Therefore, in situations of lengthy emotion work and/or management, “business-as-usual” could continue at work. Gendered patterns show that because providers of emotional support were female, women were more subject to emotion work. Men, on the other hand, seemed more reluctant to talk about private matters with persons other than their partners.

## Discussion

In our study, the focus was an exploration of reasons for sick leave due to CMDs in relation to work and private life, from the perspective of employees and managers. Our findings illustrate multiple transactions in the employees’ everyday life that were experienced as contributing to the onset and the acceleration of symptoms that eventually led to sick leave. The participants’ reasons for sick leave due to CMDs could therefore be understood as related to accumulated events situated in different social, cultural, and societal contexts of everyday life. In line with previous research, our findings relate sick leave du to CMDs to work and private life demands (cf., Nilsen et al., 2017; Svedberg et al., 2018; De Vries et al., 2018), and a downward process including individual and environmental aspects (Holmgren & Dahlin Ivanoff, 2004; Verdonk et al., 2008). Using a transactional perspective, and thus positioning the experience of problematic dimensions of everyday life in relation to the workplace context and social norms of work and gender, aids a further understanding of risks for sick leave due to CMDs. The discussion will focus on the following risks: a) unawareness of social norms of work and gender in a workplace setting; b) a greater share of practical, mental, and emotional burdens for women; and c) lack of transparency about symptoms of CMDs at the workplace, more pronounced among men. Our results can contribute to an increased awareness of the integrated effect of matters in work and private life and the influence of social norms on worker health. Awareness on an organizational level is of importance to manage situations when employees are at risk of entering sick leave.

Gendered aspects of work and sick leave can be understood as something both pervasive yet elusive. The implication of gender on employees’ work life is previously described as challenging to capture in qualitative research (Flinkfeldt, 2017; Vidman, 2007). In our study, the influence of high workload and boundaryless work on employee health was described similarly among participants. Gender segregation, or the influence of gender norms at work, were rarely explicitly mentioned when the employees and managers described the reasons for sick leave. As such, both managers and employees seemed less aware of gender-related aspects in a workplace setting or between work sectors, than in relation to private life. Consequently, few equality policies or adjustments were in use. Somewhat paradoxically, managers often said that they strived for a gender-balanced workplace but also stated that gender rarely was significant at work. The contradiction of saying that gender is not so important in the workplace, and then maintaining that gender is important (by striving for gender balance) has previously been identified by Magnusson and Marecek (2012) as something that gives high merit to individualism. Because of the greater awareness of gender inequalities in private life, managers regarded flexible work schedules as a gender equality policy and mentioned few other measures. Flexible schedules entailed the opportunity to work late evenings but hindered opportunities for recovery. Moreover, flexible schedules indicated a narrow definition of gender equality and implied a certain risk of perpetuating social norms of work and gender. Although flexible working hours were appreciated, it is important to note that they can contribute to maintaining worker/parent ideals that are hard to achieve and influence the employee’s well-being. Focusing exclusively on flexible working hours risks excluding other gender equality measures at work and reproducing the idea of women as primarily carers/mothers (Pereira, 2021).

Our findings show that one set of reasons which gave rise to sick leave was the practical, mental, and emotional burden carried by women, in line with findings from recent studies of work and home-related aspects behind sick leave due to CMDs and RTW (Nilsen et al., 2017; Nybergh et al., 2020). By comparing the processes as described by men and women we argue that “doing gender” for women encompassed more responsibility for both family-related work and emotion work in a wider setting, both before and during sick leave. In line with previous research (Nybergh et al., 2020), our study shows that doing gender for men in family life meant having a certain leeway when on sick leave. Moreover, the situation for women in our study is in line with research that has focused not only on time spent on household work but also on caring for others (Cunha et al., 2016; Flinkfeldt, 2017; Hjálmsdóttir & Bjarnadóttir, 2020). Women’s unequal position in terms of caring for others, providing emotional support, and doing emotion work also made them vulnerable to challenging life events. These could either come on top of an already heavy workload or run as a parallel long-term process in a ”business-as-usual way”. In both cases, they could negatively affect employees’ sense of “mastery of life”, which has been identified as a central aspect of RTW-processes for women (Holmgren & Dahlin Ivanoff, 2004). Hence, managing a larger share of burdens in private life for women is embedded in and shaped by contextual circumstances. Given nearly equal labour market participation among men and women (European Institute for Gender Equality (EIGE), 2020), the increase of high strain jobs and risk for sick leave identified in female dominated sectors (Aronsson et al., 2021; Lidwall et al., 2018), the situation for women entails risks of dual burdens over long periods.

In our study, the negative effects on mental health for men were more apparent in their stories about work. Just like female employees, men described a strategy of struggling at work when symptoms of CMDs arose but also powerlessness related to their impaired work performance. Previous research point to the specific challenges for men, related to social norms of masculinity, when experiencing lost functions due to CMDs (Drioli-Phillips et al., 2020), and maintaining masculine ideals through their avoidance of talking about emotional distress (Drioli-Phillips et al., 2020; Scholz et al., 2017). In our study, a reluctance to disclose symptoms of CMDs at work was seen among both men and women. However, while women in our study exemplified having a wider network to discuss private matters, among the male participants it was evident that their main support was their partners. For men, the powerlessness they felt at work could lead them to ruminate about workplace problems, and maintain a hard jargon in destructive work environments, which in turn increased their distress. The struggles in men’s lives might therefore be less obvious to their managers and certain gender blindness in work settings may make it difficult to identify situations that men find unfavourable. Men’s avoidance to disclose their symptoms, found in this and other studies (Drioli-Phillips et al., 2020), could be an additional explanation of the gender gap in sick leave due to CMDs.

The dynamic and ongoing nature of what people do and experience in their work and private life, and how this contributes to a person’s health (cf., Fritz & Cutchin, 2017; Wilcock & Hocking, 2015), can be useful in understanding the downward process leading to sick leave due to CMDs shown in our and other studies (Holmgren & Dahlin Ivanoff, 2004; Verdonk et al., 2008). Barriers and resources at work, including organizational and managerial levels, are found central for sustainable work (K. Nielsen et al., 2018). On an organizational level, it is relevant to recognize the negative impact of high workload and boundaryless work, but also the possible downsides for the employee to disclose their situation if worker ideals prevail. Moreover, it is reasonable to believe that there is a window of opportunity for preventing sick leave between early symptoms of CMDs and reporting sick. Earlier, the need for effective communication (Holmgren & Dahlin Ivanoff, 2007), sensitivity to privacy and disclosure, and competence in managing conflicts (Johnston et al., 2015) on a managerial level is highlighted. A better understanding of the risks of sick leave may help to identify unhealthy situations at work and adjust them. To enable sustainable working lives, we argue that it is relevant to take the many transitions that occur during the life course into account. These include such life events as having small children, changing jobs, and experiencing emotionally demanding situations in private life. From a gender perspective, our findings indicate the importance of solutions besides flexible working arrangements, both on a societal and an organizational level. To advance this knowledge, future research into worker health should explore possible ways to increase organizational awareness of social norms of work and gender. Such research may well focus on an in-depth exploration of gendered differences in various work sectors and how these relate to sick leave.

### Strengths and limitations

Trustworthiness in a qualitative study relates to the integrity of data, a balance between participants’ meaning and research interpretation, and the communication of findings (Williams & Morrow, 2009). In our study, the inclusion of participants with diverse characteristics and viewpoints created preconditions for a rich data set, for example, variations in age, educational level, work sector, and gender. Possible limitations are related to the fact that the interviews had to be conducted remotely due to the COVID-19 pandemic, and recall bias. Moreover, in line with others (Flinkfeldt, 2017; Vidman, 2007), we experienced challenges to achieve a comprehensive understanding of gendered experiences in workplace settings, for example, experiences of gender segregation or differences between work sectors. Rather, participants described themselves and others in gender-neutral terms as either a collective of humans (sharing common experiences and preferences) or individuals (having specific experiences and preferences). It is likely that a different design, with a focus on specific work sector/s, would facilitate a deeper understanding of gender differences at work coupled with risks of sick leave due to CMDs.

To elicit detailed and authentic responses and to situate participants’ experiences through context several strategies were used. Information about confidentiality was given on several occasions. The participants were encouraged to choose a space where they could speak freely, and follow-up questions and prompts were used. Moreover, the participants were asked to illustrate their experiences with stories or cases (Bergen & Labonté, 2020). In analysis, the theoretical perspectives of occupation and gender enabled us to obtain a richer understanding of person-situation relations in work and private life, as well as latent meanings related to social norms of work and gender. The involvement of a multidisciplinary team of researchers was helpful to support each other in a reflexive process. The detailed description of our theoretical approach, the setting for the study, and the design used make it easier for the reader to assess the trustworthiness and judge the transferability of findings.

### Conclusion

The present study builds on employees’ and managers’ experiences of reasons for sick leave due to CMDs in relation to both work and private life. Respondents understood sick leave as related to accumulated events situated in different social, cultural, and societal contexts of everyday life. The findings show that problems like 1) struggling with high workload and boundaryless work, 2) struggling with feelings of insecurity and lack of support at work, along with 3) managing unequally shared practical, mental, and emotional work in private life, seem to generate and reinforce symptoms of CMDs. Policies and practices to enable healthy work environments and a sustainable working life should aim for awareness of situations of risk as well as a recognition of the impact of social norms on the employee. Moreover, organizations should encourage a transparent dialogue between employees and managers and create space for the many transitions in everyday life that take place during working life.

## Data Availability

Data is available upon reasonable request.
